# Intensified Neuronal Investment in the Processing of Chemosensory Anxiety Signals in Non-Socially Anxious and Socially Anxious Individuals

**DOI:** 10.1371/journal.pone.0010342

**Published:** 2010-04-23

**Authors:** Bettina M. Pause, Katrin Lübke, Joachim H. Laudien, Roman Ferstl

**Affiliations:** 1 Department of Experimental Psychology, University of Duesseldorf, Duesseldorf, Germany; 2 Department of Psychology, University of Kiel, Kiel, Germany; University of Granada, Spain

## Abstract

**Background:**

The ability to communicate anxiety through chemosensory signals has been documented in humans by behavioral, perceptual and brain imaging studies. Here, we investigate in a time-sensitive manner how chemosensory anxiety signals, donated by humans awaiting an academic examination, are processed by the human brain, by analyzing chemosensory event-related potentials (CSERPs, 64-channel recording with current source density analysis).

**Methodology/Principal Findings:**

In the first study cerebral stimulus processing was recorded from 28 non-socially anxious participants and in the second study from 16 socially anxious individuals. Each individual participated in two sessions, smelling sweat samples donated from either female or male donors (88 sessions; balanced session order). Most of the participants of both studies were unable to detect the stimuli olfactorily. In non-socially anxious females, CSERPs demonstrate an increased magnitude of the P3 component in response to chemosensory anxiety signals. The source of this P3 activity was allocated to medial frontal brain areas. In socially anxious females chemosensory anxiety signals require more neuronal resources during early pre-attentive stimulus processing (N1). The neocortical sources of this activity were located within medial and lateral frontal brain areas. In general, the event-related neuronal brain activity in males was much weaker than in females. However, socially anxious males processed chemosensory anxiety signals earlier (N1 latency) than the control stimuli collected during an ergometer training.

**Conclusions/Significance:**

It is concluded that the processing of chemosensory anxiety signals requires enhanced neuronal energy. Socially anxious individuals show an early processing bias towards social fear signals, resulting in a repression of late attentional stimulus processing.

## Introduction

Within all major taxa stress responses to danger are associated with the release of chemical stress signals, which induce physiological stress adaptations within surrounding conspecifics [1]–[6]. Different sensory systems seem to be specialized to process chemosensory stress signals in mammals (the main olfactory system, trace-amine-associated receptors, the vomeronasal organ, Grueneberg ganglion cells [see 7–10]).

In humans, the processing of chemosensory anxiety signals in the insula, precuneus, cingulate cortex, and in the fusiform cortex [11] has been discussed to resemble a contagion of the feeling of anxiety between the signal sender and the signal perceiver. However, the chemical communication of an extreme level of psychological and physiological stress (first time sky diving) results in a rather restricted activation of the amygdala [12]. Furthermore, in the context of chemosensory stress signals, the perceptual acuity for social safety cues is reduced [13], whereas the perceptual acuity for social cues of danger is increased [12], [14]. On a behavioral level, chemosensory stress signals of conspecifics augment defensive reflexes (startle) in humans [15], [16] and rats [17], [18]. However, the attentional capacities for the identification of sweat stimuli donated by anxious subjects appear to be limited [19], [20].

Very recently it has been shown, that the priming of withdrawal reflexes in the context of chemosensory anxiety signals is intensified in non-clinical socially anxious participants [15]. Thereby, it is suggested that socially anxious people might process such signals with a stronger neuronal investment than non-socially anxious people. As it is generally agreed that social phobia is associated with a bias in the processing of social information [21], an intensified neuronal processing of social fear signals might be highly disorder-specific [22].

In the present study, axillary sweat served as the anxiety signal and was collected from 49 students (28 males) while awaiting an oral examination at the university. The chemosensory control stimulus was composed of a sweat sample from the same participants while participating in an ergometer training. Upon completion of collection, all sweat samples were pooled with regard to the respective donation conditions and the donor's sex. Each of the four final homogenized samples was divided into small portions of 0.4 g and stored at −20°C. For the EEG data recording, the small portions were filled into the glass bottles of the olfactometer and renewed after each experiment. In detail, the sweat donors and the sampling procedure are described elsewhere [11].

The aim of the first experiment was to investigate in a highly time-sensitive manner (analyzing chemosensory event-related potentials; CSERPs) whether and how chemosensory anxiety signals are processed by the brain. In the second experiment non-clinical highly socially anxious participants were investigated. In order to increase the statistical power of this first time-sensitive investigation of neuronal processing of anxiety sweat, the first experiment was analyzed independently of the second experiment. However, as a result, it will not be possible to directly compare the CSERPs of non-socially anxious and socially anxious participants. It was hypothesized that chemosensory anxiety signals in general are processed advantageously by the human brain (experiment 1). In addition, the processing of chemosensory anxiety signals in socially anxious participants should resemble their attentional bias towards potential social threat (experiment 2).

## Methods

### Study 1: Non-socially anxious participants

#### Participants

Twenty-eight right-handed participants (16 males) were investigated. They were on average 24.7 years of age (SD = 4.3, range  = 19–38). As there are differences in the chemosensory perception of self and non-self [23], only those participants were selected who did not previously act as sweat donor. None of the participants suffered from any physical (self-report) or mental disease (as assessed with the Structured Clinical Interview for DSM-IV, SKID, German Version; [24]), and none reported using chronic or acute medication. All participants scored low in social anxiety (M = 11.07, SD = 3.30, according to the Social Interaction Anxiety Scale, SIAS; [25]). Participants who described themselves as medium or high socially anxious (SIAS >16) were excluded from the study. In addition, the participants scored low in depression (M = 3.50, SD = 3.33, according to the Beck Depression Inventory, BDI, German Version; [26]) and reported a medium interest in social activities (M = 2.59, SD = 0.46, according to the agreeableness scale of the Big Five personality inventory, NEO-FFI; [27]). All of them reported to be non-smokers and to be of European origin. All female participants had a regular menstrual cycle (+/− 3 days). All participants gave written, informed consent and were paid for their participation. Both studies were carried out in accordance with the Declaration of Helsinki and were approved by the ethical committee of the Medical Faculty of the University of Kiel.

#### Olfactory hyposmia screening

Prior to EEG recording, all participants were screened for general hyposmia. For this purpose, the participants were requested to identify a bottle containing phenyl-ethyl alcohol [99%, Fluka, Germany, 1∶200 (*v/v*) diluted in 1,2-propanediol] in a set of three bottles, with the remaining two bottles containing the same volume of solvent (two consecutive trials). No participant had to be excluded due to general hyposmia.

#### Stimulus presentation

For the recording of detection performance, stimulus ratings, and EEG activity, the chemosensory stimuli were presented according to the method described by Kobal [28], using a constant flow, six channel olfactometer (OM6b, Burghart Messtechnik GmbH, Wedel, Germany). Both nostrils were stimulated simultaneously, and accordingly, both air streams (100 ml/s each) were controlled by separate mass flow meters. In the olfactometer, the glass tubes containing the stimuli were stored in a warm-water chamber, and the stimuli were delivered (duration  = 0.5 s) to the participants through a teflon tube. The temperature of the gas flow at the exit of the olfactometer was 37°C and the relative humidity was set above 80%. White noise of 80 dB (A) was presented binaurally over earplugs (Etymotic Research, ER3-14A), in order to prevent the participants from hearing the switching valves of the olfactometer.

#### Stimulus detection

To determine participants' detection performance of the chemosensory anxiety signal (anxiety sweat) and the chemosensory control stimulus (sport sweat), participants had to select the most intense stimulus from a series of three stimuli, with the remaining two blank odors consisting of pure cotton pad. This procedure was carried out twice. Participants who failed once to detect the chemosensory signal (the anxiety or the sport signal) were defined as non-detectors.

#### Procedure

All participants were tested individually in two separate sessions. During both sessions, they completed an identical experimental protocol, with the exception that either sweat donated by male or female persons was presented. The order of these sessions was balanced across participants.

Prior to the EEG recording, participants practiced the velopharyngeal closure technique [29]. The EEG was recorded during an olfactory oddball paradigm consisting of two blocks of 100 trials each (25 deviant chemosensory stimuli in a train of 75 standard stimuli). The stimuli were presented in pseudo-randomized order (with the first three trials being standards) for 0.5 s with an inter stimulus interval (ISI) of 9 s. In each of the two blocks, the standard stimulus was either the anxiety or the sport stimulus, with the order of these blocks counterbalanced across participants. The participants were instructed to avoid eye movements and to silently count the total number of odor presentations (deviants and standards).

#### Data Recording, Reduction and Analysis

The EEG was recorded in reference to the left ear lobe with Ag/AgCl electrodes (inner diameter 6 mm) from 60 scalp locations and the ear lobes, using an electrode cap (EasyCap GmbH, Germany). Two additional electrodes were placed near the right eye (3 cm above, inside the vertical pupil axis and 1.5 cm below, outside the vertical pupil axis) for the recording of vertical and horizontal eye movements. The impedance of the electrodes was always below 11 kΩ.

The physiological data were recorded, amplified, and filtered with the Aquire software (Version 4.2, NeuroScan Inc., Virginia, USA) using sampling rates of 200 Hz, a low-pass filter of 40 Hz (24 dB/ octave) and a 50 Hz notch filter. The ground was connected at FCz.

Offline, EEG signals were re-referenced to linked ear lobes, baseline corrected (0–1000 ms before stimulus onset), and high pass filtered (0.2 Hz, 24 dB/ octave). The data were then corrected for eye movements [30]. In addition, trials contaminated by any further artifacts (amplitudes between −50 and +50 µV) within the first 1400 ms after odor presentation were eliminated from the analysis. Subsequently, a zero phase shift digital low pass filter (Butterworth-filter, 7 Hz, 24 dB/ octave) was applied. The 60 scalp electrode positions were subdivided into nine areas, and a mean peak for each of these regions was calculated by averaging adjacent electrodes in anterior, central, and posterior areas for the left and right hemisphere as well as for midline electrodes [sagittal line: anterior (A), central (C), posterior (P); transversal line: left (L), midline (M), right (R); sagittal by transversal: AL: Fp1, AF7, AF3, F7, F5, F3; AM: Fpz, F1, Fz, F2; AR: Fp2, AF4, AF8, F4, F6, F8; CL: FT7, FC5, FC3, T7, C5, C3, TP7, CP5, CP3; CM:FC1, FC2, C1, Cz, C2, CP1, CPz, CP2; CR: FC4, FC6, FT8, C4, C6, T8, CP4, CP6, TP8; PL: P7, P5, P3, PO7, PO3, O1; PM: P1, Pz, P2, POz, Oz; PR: P4, P6, P8, PO4, PO8, O2]. In relation to the baseline period two separate peaks were differentiated within predefined latency windows (N1: 350–500 ms, P3: 700–900 ms; as the odors were perceived at the threshold level and with a low distinctiveness, it was refrained from dividing the P3 into different subcomponents [see 31]).

A five-way ANOVA was calculated [factors: Chemosensory Condition (anxiety condition, sport condition), Sex of Donor (male, female), Sex of Perceiver (male, female), Sagittal Line (anterior, central, posterior) and Transversal Line (left, midline, right)]. Subsequently, nested effects were calculated in accordance with Page and coworkers [32]. However, due to the small number of deviant stimuli and the poor signal-to-noise ratio for deviant stimuli, only CSERPs in response to standard stimuli were analyzed. An alpha level of p<0.05 was used for all statistical tests. Huynh-Feldt corrected degrees of freedom were calculated and corrected p-values are reported. The presentation of the CSERP results will focus on the effects including the chemosensory condition, and only significant results will be reported. Current Source Density (CSD) maps were calculated using a spherical spline model ([33], order of splines: m = 4, maximal degree of legendre polynominals  = 20).

### Study 2: Socially anxious participants

#### Participants

Socially anxious participants were 16 (8 male) students of the University of Kiel (mean age  = 21.94 years, SD  = 2.05, range  = 20–26). All socially anxious participants scored 22 or higher on the SIAS (M = 29.31, SD  = 6.07). However, they described themselves as not being depressed (BDI: M = 5.31, SD  = 3.20) and reported a medium tendency for being compassionate and cooperative towards others (agreeableness scale of the NEO-FFI: M = 2.45, SD  = 0.38). None of them suffered from any physical (self-report) or mental disease (SKID), and none reported using chronic or acute medication. All of them were dextrals, non-smokers and of European origin, and none of them participated previously as sweat donor. No participant had to be excluded due to general hyposmia. All participants gave written, informed consent and were paid for their participation.

#### Procedure

The procedure and analyses followed the same protocol as in experiment 1.

## Results

### Study 1: Non-socially anxious participants

#### Stimulus detection

Some participants were able to detect an odor of single sweat samples (either male anxiety, or female anxiety, or male sport, or female sport). However, no participant was able to olfactorily detect both chemosensory stimuli of both donor genders (Table 1). The detection rates did not significantly vary between the two odor conditions or the sex of the sweat donor (binomial tests), or with the sex of the perceiver (Fisher test). As the chemosensory stimuli were not detectable for most of the participants, it was refrained from analyzing any odor ratings.

**Table 1:** Odor detection performances (number/ percentages of participants who could detect single odors or combinations of odors).

Odour source	Sex of the odor donor	Number of odors	Non-anxious participants (N = 28)		Socially anxious participants (N = 16)	
			N	%	N	%
Anxiety sweat	Male	1	5	18	3	19
	Female	1	6	21	7	44
	Male and female	2	2	7	1	6
Sport sweat	Male	1	4	14	5	31
	Female	1	8	29	3	19
	Male and female	2	1	4	1	6
Anxiety and sport sweat	Male and female	4	0	0	0	0

#### CSERPs

**Figure 1:**
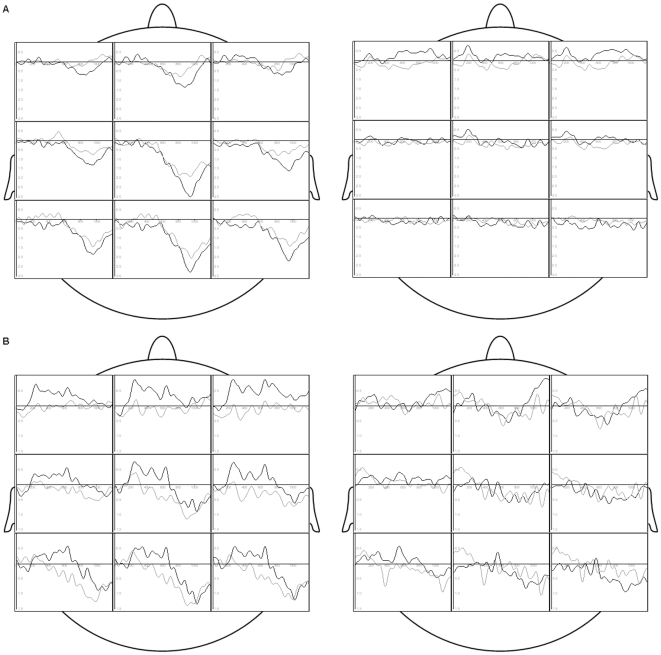
Grand Averages. (A) Grand Averages of the CSERPs of non-socially anxious female (left; N = 12, 24 sessions) and male (right; N = 16, 32 sessions) participants in response to sweat donated during the anxiety condition (black line) and the sport control condition (grey line) at pooled electrode positions (anterior left, anterior midline, anterior right, central left, central midline, central right, posterior left, posterior midline, posterior right). (B) Grand Averages of the CSERPs of socially anxious female (left; N = 8, 16 sessions) and male (right; N = 8, 16 sessions) participants in response to sweat donated during the anxiety condition (black line) and the sport control condition (grey line) at pooled electrode positions (see Fig. 1A).

In female participants the P3 peak appeared with a larger amplitude in response to chemosensory anxiety stimuli as compared to chemosensory control stimuli [Fig. 1a; Chemosensory Condition by Sex of Perceiver: F (1, 26)  = 6.30, p = 0.019, f (Cohen's f)  = 0.49, Power  = 0.67; nested effects: Chemosensory Condition in female participants: F(1, 26)  = 5.29, p = 0.030, f = 0.45, Power  = 0.60].

Male participants did not show reliable CSERPs in response to either stimulus (Fig. 1a). Accordingly, the P3 amplitude was generally larger in females than in males [Sex of Perceiver: F (1, 26)  = 10.87, p = 0.003, f = 0.65, Power  = 0.89]. This sex effect was evident at all three transversal electrode lines, but most pronounced at midline electrode positions [Sex of Perceiver by Transversal: F (2, 52)  = 7.84, p = 0.001, f = 0.55, Power  = 0.94].

The N1 component was not affected by the donation condition or the sex of the perceiver, and none of the components varied with the sex of the donor. The chemosensory condition did not affect the latency of any component.

#### CSDs

**Figure 2:**
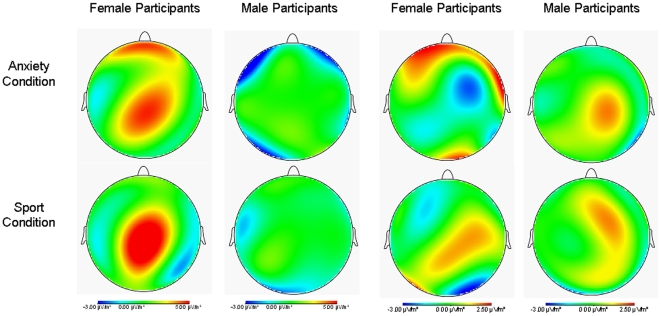
Current Source Density (CSD) maps. Neuronal processing of chemosensory anxiety signals and sport control stimului plotted as CSD maps. The two left columns show the CSDs of non-socially anxious female and male participants plotted for the time point of the maximum P3 amplitude. The two right columns show the CSDs of socially anxious female and male participants plotted for the time point of the maximum N1 amplitude. Blue colors represent a weaker magnitude (neuronal sinks) and red colors represent a stronger magnitude of CSD (neuronal sources).

At the time of the maximum P3 amplitude (805 ms–810 ms), females showed much stronger neuronal activation than males in response to both chemosensory stimuli (Fig. 2). In females, centrally located neuronal activity was related to either odor source, whereas medial frontal activation was specifically associated with the perception of chemosensory anxiety signals. The prefrontal activation appears with a left sided dominance between 400 and 600 ms after stimulus onset and reappears between 700 and 900 ms with a medial dominance. After 900 ms the frontal activity vanishes. However, the non-specific central activation can be observed 500 ms after stimulus onset and remains with slight local changes for about 1 s (see Supplementary Material, Video S1).

### Study 2: Socially anxious participants

#### Stimulus detection

As within Study 1, the chemosensory stimuli were difficult to detect. No participant was able to detect all of the four olfactory stimuli (Table 1). The detection rates did not significantly vary with the chemosensory condition, the sex of the sweat donor (binomial tests), or with the sex of the perceiver (Fisher test). As the chemosensory stimuli were not detectable for most of the participants, odor ratings were not analyzed.

#### CSERPs

The amplitude of the N1 component in socially anxious female participants was larger in response to chemosensory stimuli donated during the anxiety condition than in response to chemosensory stimuli donated in the sport control condition above posterior scalp regions [Chemosensory Condition by Sex of Perceiver by Sagittal: F (2, 28)  = 5.93, p = 0.009, f = 0.74, Power  = 0.84; nested effects: Chemosensory Condition by Sagittal within female participants: F (2, 28)  = 5.94, p = 0.009, f = 0.65, Power  = 0.84; Chemosensory Condition within female subjects within posterior electrode positions: F (1, 15)  = 5.49, p = 0.033, f = 0.61, Power  = 0.59] as well as at posterior left electrode positions [Chemosensory Condition by Sex or Perceiver by Sagittal by Transversal: F (4, 56)  = 4.22, p = 0.011, f = 0.55, Power  = 0.90; nested effects: Chemosensory Condition by Sagittal by Transversal within female participants: F (4, 56)  = 4.85, p = 0.006, f = 0.59, Power  = 0.94; Chemosensory Condition by Sagittal within female participants within left electrode positions: F (2, 30)  = 10.36, p<0.001, f = 0.83, Power  = 0,98; Chemosensory Condition by Sagittal within female participants within midline electrode positions: F (2, 30)  = 4.04, p = 0.032, f = 0.52, Power  = 0.68; Chemosensory Condition within female participants within left electrode positions within posterior electrode positions: F (1, 15)  = 10.73, p = 0.005, f = 0.85, Power  = 0.86; see Fig. 1b].

In socially anxious participants, the N1 latency was shorter in response to chemosensory stimuli donated during the anxiety condition as compared to chemosensory stimuli donated during the sport control condition [Chemosensory Condition: F (1, 14)  = 9.80, p = 0.007, f = 0.84, Power  = 0.83]. This effect was more pronounced in male than in female participants [Chemosensory Condition by Sex of Perceiver: F (1, 14)  = 6.71, p = 0.021 f = 0.69, Power  = 0.83; nested effects: Chemosensory Condition within male participants: F (1, 14)  = 16.37, p = 0.001, f = 1.08, Power  = 0.96].

The amplitude and latency of the P3 were not affected by the chemosensory condition. The sex of the odor donor did not affect either component.

#### CSDs

At the time point of the maximum N1 amplitude (435–440 ms after valve activation), socially anxious female participants show stronger brain activations across left and right frontal scalp areas in response to chemosensory anxiety signals than in response to the control stimuli (Fig. 2). The frontal activity starts about 300 ms with a right sided maximum, and about 400 ms after stimulus onset with an additional left sided maximum. The frontal activity vanishes briefly at about 500 ms after valve activation and reappears between 500 and 700 ms with a medial maximum (see Supplementary Material, Video S2). During the entire time period of the CSERP no frontal neuronal sources can be detected in socially anxious females smelling sport sweat. Instead, the chemosensory control stimuli are processed by centrally located neocortical brain areas, between 400 and 600 ms after valve activation (Fig. 2).

## Discussion

### Study 1: Non-socially anxious participants

The EEG data reveal that the processing of chemosensory anxiety signals engages significantly more neuronal resources than the chemosensory processing of sport sweat. Thereby, the results are in line with recent brain imaging studies [11], [12], demonstrating that the processing of chemosensory anxiety or stress signals requires more neuronal resources than the processing of body odor signals sampled in a non-emotional control condition. While the chemosensory stimuli used in the brain imaging studies were perceived to have a weak odor, most of the participants in the present experiment could not detect an odor when presented with the sweat samples. Therefore, the present study strongly supports the conclusion drawn by Mujica-Parodi et al. [12] and Prehn-Kristensen et al. [11], that the neuronal processing of chemosensory anxiety signals is not consciously mediated.

The processing of axillary odors unequivocally recruited stronger neuronal activity in females than in males. The intense neuronal processing of body odor signals in females was accompanied by a differential response to the two chemosensory stimuli within the P3 latency range. So far, two studies reported females to respond more sensitively than males to chemosensory anxiety signals [13], [20], whereas other studies did not find any gender differences [11], [12], [15]. However, no study described a processing advantage for chemical signals of emotions in male participants. Even though a larger late positivity within the ERP in females has been observed in response to common odors [34] and socially relevant information (facial expressions of emotions; [35]), null effects of gender in emotional stimulus processing have also been reported (odors: [36]; emotional stimuli: [37]). Here, it is postulated that sex effects in the processing of emotional stimuli are most pronounced for social emotional stimuli [38] and most importantly, for emotional stimuli with a weak perceptional salience [39], [40]. In accordance with this assumption, the stimuli administered in the present study were perceived subliminally by most of the participants. A comparable strong effect of gender was only found for the perception of subliminally presented facial expressions in the context of chemosensory anxiety signals [13].

Within the P3 latency range, females showed neuronal activity in response to both body odors above central brain areas. Additional medial frontal activation predominantly occurred in response to the anxiety signals. Recently, it was demonstrated by CSD analysis that neuronal activity located in medial frontal brain areas is most prominent in the P3 latency window and in response to potentially harmful odors [41]. In general, medial prefrontal activation is the most common observation in emotional activation studies [42] and may be related to flexible physiological adjustments in (socially) relevant situations [43], as well as to the integration of sensory and cognitive information in order to adjust physiological activity [44].

### Study 2: Socially anxious participants

Even though most of the socially anxious participants could not smell the chemosensory stimuli, the processing of anxiety-related chemosignals was faster and recruited more neuronal resources than the processing of sport-related chemosignals. Similar to non-socially anxious participants, the large potentials in response to chemosensory anxiety signals could be observed in female participants only. However, the faster processing of chemosensory anxiety signals was more pronounced in males.

Individuals scoring high in social phobia engage neuronal investment in the processing of chemosensory anxiety signals at an earlier processing level (N1) than non-socially anxious participants (P3). It has repeatedly been reported that social anxiety is characterized by a bias towards social and threat related information at an early level of information processing. Especially the P1 component of the visual ERP is increased in socially anxious participants during the processing of human faces [45], [46]. This processing advantage occurs most distinctly in response to negative or angry facial expressions [47], [48]. It is in line with the present study that the early processing advantage for negative social stimuli in social phobia patients is accompanied by a reduced late stimulus processing [46]. Hereby, it is indicated that attentional avoidance follows the initial orientation towards negative social information.

It has repeatedly been reported that the processing of neutral (e.g. [49]), negative (e.g. [50]), or angry faces (e.g. [51]) in social phobia requires an increased neuronal activity within the amygdala. However, just recently it could be shown that the increased amygdala activity seems rather to be related to the processing of angry than of fearful faces, and does not differentiate between generalized anxiety and social phobia [22]. In contrast, patients with social phobia but without generalized anxiety recruit more neuronal resources during the processing of fearful faces, especially in frontal brain regions (middle frontal gyrus/frontal polar cortex, BA 10; lateral frontal cortex, BA 46). The CSD maps of the present study indicate that socially anxious individuals engage similar brain circuits during the processing of chemosensory anxiety signals. However, in the present study, the degree of general anxiety was not obtained and therefore, could be confounded with social anxiety. Instead, as socially anxious and non-anxious participants scored low in depression and medium in social interest, it was excluded that the present effect of social anxiety is biased by the degree of depression or social interest.

### General discussion

In combination, both studies demonstrate that distinct emotional states, like anxiety, are communicated chemosensorily. Especially in females, the processing of chemosensory anxiety signals requires more neuronal activity than the processing of body odor donated in an emotionally neutral condition. In socially anxious males, the processing of anxiety related chemosignals is faster than the processing of the control stimuli. Thus, the here reported results are in line with previous studies, indicating a chemosensory transmission of anxiety or stress-related experience in humans [11], [12], [14]. Most importantly, the present study could demonstrate that understanding the phenomenon of chemosensory communication of anxiety may have important applied consequences. Participants scoring high in social anxiety are at risk to develop social phobia, one of the most common anxiety disorders, with a lifetime prevalence of 12.6% [52]. As social phobia is a powerful risk factor for subsequent depressive illness and substance abuse [53], the explanation of its pathogenesis is of special importance. In the present study, socially anxious participants showed a processing advantage for chemosensory anxiety signals already at a very early level of stimulus processing. Therefore, in the future, this knowledge could gainfully be integrated into behavioral therapy of social anxiety.

It should be noted, that the effects reported here could be demonstrated even though the chemosensory stimuli were applied repeatedly (200 times) and with relatively short ISIs (9s) in each EEG session. Repeated odor stimulation would result in a strong habituation and thus a strong reduction of the CSERP amplitudes [28], [54]. However, recent research indicates that chemosensory alarm signals are not processed in olfactory, but in separate sensory systems [8], [10]. Accordingly, it has been reported that the response to social chemosignals is less prone to effects of habituation than the response to common odors [55]. For example, rodents respond to a continuous exposure to chemosensory alarm signals of consepecifics with a 40 min lasting autonomic stress response (increase in body temperature [56]).

Finally, as only anxiety related signals were investigated in the present study, it can not be ruled out whether the here reported effects are emotion specific or related to the perception of social distress signals in general. More studies are needed, exploring as to whether other basic emotions like anger, disgust or happiness chemosensorily induce specific physiological adaptations in the perceiver. In sum, the research on chemosensory communication of emotions may broaden the knowledge about phylogenetically ancient emotions in humans, offering a new method to define basic emotions in humans and understanding emotion related disorders.

## Supporting Information

Video S1 Time course (0–1200 ms after valve activation) of the current source density distribution in non-socially anxious females (N = 12), perceiving chemosensory anxiety signals from male and female donors. Blue colors represent a weaker magnitude (neuronal sinks) and red colors represent a stronger magnitude of CSD (neuronal sources). Left sided, the voltage distribution is plotted as a grand average at Cz across the same female participants.
(1.16 MB MP4)

Video S2 Time course (0–1200 ms after valve activation) of the current source density distribution in socially anxious females (N = 8), perceiving chemosensory anxiety signals from male and female donors. Blue colors represent a weaker magnitude (neuronal sinks) and red colors represent a stronger magnitude of CSD (neuronal sources). Left sided, the voltage distribution is plotted as a grand average at Cz across the same female participants.
(1.30 MB MP4)
